# Publication bias in animal research presented at the 2008 Society of Critical Care Medicine Conference

**DOI:** 10.1186/s13104-017-2574-0

**Published:** 2017-07-07

**Authors:** Una Conradi, Ari R. Joffe

**Affiliations:** 1 Faculty of Science, University of Alberta, Edmonton, Canada; 2 Faculty of Medicine, Department of Pediatrics, Stollery Children’s Hospital, University of Alberta, Edmonton, AB Canada; 3 John Dossetor Health Ethics Center, University of Alberta, Edmonton, AB Canada; 4 4-546 Edmonton Clinic Health Academy, 11405 87 Avenue, Edmonton, AB T6G 1C9 Canada

**Keywords:** Animal experimentation, Bias (epidemiology), Intensive care, Publication bias

## Abstract

**Background:**

To determine a direct measure of publication bias by determining subsequent full-paper publication (P) of studies reported in animal research abstracts presented at an international conference (A).

**Methods:**

We selected 100 random (using a random-number generator) A from the 2008 Society of Critical Care Medicine Conference. Using a data collection form and study manual, we recorded methodology and result variables from A. We searched PubMed and EMBASE to June 2015, and DOAJ and Google Scholar to May 2017 to screen for subsequent P. Methodology and result variables were recorded from P to determine changes in reporting from A. Predictors of P were examined using Fisher’s Exact Test.

**Results:**

62% (95% CI 52–71%) of studies described in A were subsequently P after a median 19 [IQR 9–33.3] months from conference presentation. Reporting of studies in A was of low quality: randomized 27% (the method of randomization and allocation concealment not described), blinded 0%, sample-size calculation stated 0%, specifying the primary outcome 26%, numbers given with denominators 6%, and stating number of animals used 47%. Only being an orally presented (vs. poster presented) A (14/16 vs. 48/84, p = 0.025) predicted P. Reporting of studies in P was of poor quality: randomized 39% (the method of randomization and allocation concealment not described), likely blinded 6%, primary outcome specified 5%, sample size calculation stated 0%, numbers given with denominators 34%, and number of animals used stated 56%. Changes in reporting from A to P occurred: from non-randomized to randomized 19%, from non-blinded to blinded 6%, from negative to positive outcomes 8%, from having to not having a stated primary outcome 16%, and from non-statistically to statistically significant findings 37%. Post-hoc, using publication data, P was predicted by having positive outcomes (published 62/62, unpublished 33/38; p = 0.003), or statistically significant results (published 58/62, unpublished 20/38; p < 0.001).

**Conclusions:**

Only 62% (95% CI 52–71%) of animal research A are subsequently P; this was predicted by oral presentation of the A, finally having positive outcomes, and finally having statistically significant results. Publication bias is prevalent in critical care animal research.

**Electronic supplementary material:**

The online version of this article (doi:10.1186/s13104-017-2574-0) contains supplementary material, which is available to authorized users.

## Background

Publication bias (PB) refers to preferential publication of research findings that have statistically significant positive outcomes [1]. This is problematic because the published literature is what scientists and clinicians use to inform their research and clinical practice. Indeed, the enterprise of evidence based medicine rests on the assumption that the published literature is an accurate representation of the current knowledge base. If evidence from systematic literature reviews is a biased representation of knowledge, this is a heavy blow to the paradigm of evidence based medicine [2]. The problem of PB is well recognized in clinical research; reviews suggest that only about half of research is published, and positive findings are a strong predictor of publication [3, 4].

Biomedical animal research (AR) is used to inform human research and practice on the assumption that animals are causal analogical models of human physiology and response to drugs and disease [5]. If PB is prevalent in AR, this is problematic for several reasons. First, evidence from published AR would be a biased representation of pre-clinical knowledge and thus be potentially misleading and dangerous for informing human medicine [6, 7]. Second, the moral permissibility of AR, based on the claim that the harm to sentient animals used in AR is outweighed by large human benefit from this AR would be unacceptably weakened [8]. For example, animals harmed in unpublished AR cannot contribute to biomedical knowledge, and thus were harmed for no good reason. Moreover, the animals used in unpublished AR that had negative findings were harmed with additional potential harm also to humans, due to subsequent biomedical research and practice following false leads or safety data from the published AR [9]. Some data indirectly suggests PB in AR is common. Using statistical tools that examine small study bias (a larger effect size in small studies compared to larger studies), several AR reviews suggest PB occurs [10–17]. Another recent study found AR suffers from excess significance bias, again indirectly suggesting PB [18]. Of 174 basic science study abstracts submitted to a major gastroenterology conference, of which 90 were “animal studies”, subsequent publication occurred for 47%, with no statistically significant predictors of publication found; however, results for AR were not reported separately [19]. To our knowledge there has not been a direct assessment of whether PB occurs in AR.

Here we aimed to determine whether AR abstracts presented at an international critical care conference are subsequently published in the peer-reviewed literature, and what the predictors of subsequent publication may be. We chose critical care because inducing critical illness may be one of the most invasive fields of AR. In addition, our method allows a direct assessment of PB of AR that has reached the stage of warranting presentation to peers.

## Methods

### Ethics statement

The University of Alberta Health Research Ethics Board waived the requirement for review or consent because the study involved only publicly available data, and no individual that would require consent to participation.

### Abstract review

We reviewed 100 random published abstracts of AR from the 2008 Society of Critical Care Medicine international conference [20]. The abstracts were chosen using a random number generator. There were no restrictions other than that the abstract reported an AR experiment, defined as: a procedure for collecting scientific data on the response to an intervention in a systematic way to maximize the chance of answering a question correctly or to provide material for the generation of new hypotheses [21]. If there was any doubt about inclusion, this was discussed among the two authors to achieve consensus. A data collection form and instruction manual (see Additional files 1, 2) were created based on published Canadian (the Canadian Council on Animal Care in science guidelines “Choosing an appropriate endpoint in experiments using animals for research, teaching and testing” and “Animal use protocol review”), United States (the Institute for Laboratory Animal Research, National Research Council “Guidance for the description of animal research in scientific publications”), and United Kingdom (the National Center for the 3Rs “Animal Research: Reporting of In Vivo Experiments” ARRIVE guidelines) recommendations for reporting AR [22–25]. These guidelines were used as they are comprehensive, well referenced, readily available, and based upon literature review. For example, the ARRIVE guidelines were developed to improve the quality of reporting AR, and are endorsed by over 100 journals from all over the world [22]. Data were obtained for factors important to methodological quality.

The data collection form was completed for all 100 critical care AR (using mammals) abstracts. Both authors independently completed forms for the first 10 abstracts, discussing the data after every fifth abstract until consistent agreement was obtained. Thereafter, one author completed forms on all abstracts, and the other author independently did so for every fifth abstract (with discussion of the data to maintain consistent agreement), and for any data considered uncertain (with discussion until consensus). The instruction manual made clear definitions for all data collection; for example, a sample size calculation was defined as describing, for the primary outcome, a p value (alpha), power (1-beta), and minimally important difference (the difference between groups that the study is powered to detect).

### Search for publication

After the abstract data was finalized in the data collection form, we searched both PubMed and EMBASE to determine subsequent publication of the data presented in the abstracts. The search strategy was defined in the instruction manual. We searched for the first, second, or last author; and at least two MeSH subject terms (e.g., acute lung injury, or sepsis). The search strategy was developed to be as inclusive as possible, by using the operator “OR” between the authors searched and between the MeSH terms used. Abstracts that were later published as only a small part of a larger study were considered published. All titles and abstracts of identified publications were reviewed to determine if they reported the study from the abstract. To be considered publication of the abstract results the published paper needed to report the same experiment, and we included the publication even if more animals were used and/or more experiments were done than reported in the abstract. If there was any doubt, both authors together discussed the titles and abstracts and reached consensus. If publication could not be found by one of the authors, the second author also independently searched to confirm this; if publication was then found, both authors discussed the finding and reached consensus. The search was done from 2007 (up to 14 months prior to the conference date) to June 2015 (up to 86 months after the conference date) inclusive.

In response to an anonymous reviewer, in May 2017 one author (ARJ) searched DOAJ (the Directory of Open Access Journals) and Google Scholar for subsequent publication of any of the abstracts determined unpublished by the above strategy. The search strategy was modified to be consistent with the PubMed and EMBASE searches. On DOAJ we searched separately for each of the first or last author, and if more than 30 publications were listed, we added (separately, one at a time) each of two subject terms from the abstract title. On Google Scholar we searched for each of the first or last author, added (separately, one at a time) each of two subject terms from the abstract title into the “contains all of the words” box, and limited the search to from years 2007 to 2017. We screened all returned titles, and if necessary, abstracts and full text, up to and including 5 pages of each search result.

Finally, as suggested by another reviewer, we emailed an author of each unpublished abstract to ask if the abstract had been published in full, and if so, to provide the citation. For each abstract, one author (ARJ) searched PubMed for the abstract author (starting with first author, and if no email found, last author, second author, second last author, etc.) in order to obtain a corresponding author email address. To be sure this was a current email for the correct abstract author, the address was to be from a publication since 2010, and on a topic related to the abstract, preferably with a same coauthor(s) as in the abstract. If the email was returned undeliverable, the search was repeated to obtain another of the abstract authors’ email. For the 3 emails that remained undeliverable we identified a current corresponding co-author of a publication with the target author, and asked this contact to forward the request.

### Publication review

A data collection form and instruction manual (see Additional files 1, 2) were used for each identified publication. Both authors independently completed data collection forms for the first 5 identified publications, discussing the data until consistent agreement was obtained. Thereafter, one author completed forms on all publications, and the other author independently did so for every fifth publication (with discussion of the data to maintain consistent agreement), and for any data considered uncertain (with discussion until consensus). The instruction manual made clear definitions for all data collection variables.

### Statistics

Data are presented using descriptive statistics, and were analyzed using SPSS. The primary outcome was pre-specified as subsequent publication of AR abstracts, with 95% adjusted Wald Confidence Intervals (CI). Assuming the subsequent publication rate of AR will be similar to that for clinical research weighted mean full publication rate of 44.5% (95% CI 43.9–45.1%) [3], an abstract sample of n = 80 will provide reasonable 95% CI of ±10%. Thus, we decided to include 80 abstracts, and if the timing allows, add a random sample of 20 abstracts to increase the sample size prior to any data analysis. We compared abstracts that were published to those that were not using Chi square and Fisher’s Exact test, with statistical significance accepted at p < 0.05 without correction for multiple comparisons. Pre-defined possible predictors included methodology (randomization, blinding, sample size calculation, primary outcome specification, results reported with denominators, number of animals used stated), ethical (number of animals used >19, highest species used rodent vs other), outcome (positive finding, statistically significant finding), and type of animal model (sepsis, drug used, surgery performed, animals stated to be killed) variables. Post-hoc we tested for possible predictors including methodology (randomization, blinding) and outcome (positive finding, statistically significant finding) variables updated with information from publication if available. We also determined whether there were changes in important information reported between the abstract and subsequent publication, using our a priori definitions in the data collection form and instruction manual.

## Results

### Primary outcome

61 [61% (95% CI 51–70%)] abstracts were subsequently published after a median 19 [IQR 9–33, range 0–68] months by searching PubMed and EMBASE. The search of DOAJ found no publications, and Google Scholar found one further publication, for a publication rate of 62% (95% CI 52–71%). Publication was usually within 3 years; months to publication was mean 22.6 (SD 17.1), median 19 [IQR 9, 33.3], range 0–68 months; the 80th percentile was 35 months (2.9 years); 90th percentile 52 months (4.3 years); and 95th percentile 60 months (5 years).

Emails were sent on May 25 (and to non-responders again on June 1) to an abstract author for the initially determined 39 unpublished abstracts. There were 13 replies by June 8 (within 2 weeks): 9 confirmed non-publication, and 4 claimed publication. However, the citations provided in the 4 clearly did not match the abstract methodology despite being on a similar topic (1 was published 4 years before the abstract and used different study interventions; 1 was a global head injury model whereas the abstract was of a focal brain injury model; 1 randomized to different intervention groups than in the abstract; and 1 was published 12 years before the abstract and used a different animal model). Thus, no publication was identified by this emailing strategy.

### Predictors of abstract publication

The differences between published and unpublished abstracts are given in Table 1. Methodological quality of reporting in the abstracts was poor, with randomization in 27 (27%; none reported the method of randomization or allocation concealment), blinding in 0 (0%), sample size calculation in 0 (0%), primary outcome stated in 26 (26%), number with denominators given for main outcomes in 6 (6%), and number of animals used stated in 47 (47%; median 18, IQR 11–24, range 1–60; total 957). Most abstracts reported mainly positive outcomes (90, 90%), and these outcomes were statistically significant in 55 (55%). The only statistically significant predictor of subsequent publication was being an oral presentation at the conference (p = 0.024).

**Table 1:** Potentially predictive variables for subsequent publication of abstracts presented at an international critical care conference

Potential predictor variable	Published (n = 62)	Non-published (n = 38)	p value^a^
Type of presentation			
Oral (vs. poster) presentation	14 (23%)	2 (5%)	0.025
Research location in North America^b^	40 (65%)	31 (82%)	0.075
Methodological quality variables			
Randomized	17 (27%)	10 (26%)	0.99
Blinded	0 (0%)	0 (0%)	–
Primary outcome given	13 (21%)	13 (34%)	0.16
Numbers with denominators	4 (6%)	2 (5%)	0.99
Number of animals stated	30 (48%)	17 (45%)	0.84
Sample size calculation	0 (0%)	0 (0%)	–
Ethical quality variables			
Highest species rodent^c^	35 (56%)	26 (68%)	0.23
>19 animals used	15/30 (50%)	5/17 (29%)	0.14
Outcome variables			
Main outcomes positive	57 (92%)	33 (87%)	0.50
Statistically significant result	35 (56%)	20 (53%)	0.71
Type of animal model			
Sepsis	27 (44%)	13 (34%)	0.41
Drug used	24 (39%)	15 (39%)	0.99
Surgery performed	25 (40%)	13 (34%)	0.67
Animals stated to be killed	34 (55%)	21 (55%)	0.99

Potential predictor variable	Data in publication (n = 62)	Data in abstract (n = 38)	p value
Post-hoc comparisons			
Indicators of publication bias			
Main outcomes positive	62 (100%)	33 (87%)	0.003
Statistically significant result	58 (94%)	20 (53%)	<0.001
Indicators of methodological quality			
Randomized	24 (39%)	10 (26%)	0.20
Blinded	4 (6%)	0 (0%)	0.080

^a^ Comparisons made using Fisher’s Exact or Chi square test

^b^ Asia 17 (17%); North America 71 (71%), Europe 15 (15%); Australia/New Zealand 1 (1%), and Africa or South America 0

^c^ Species used were: rodent (61), rabbit (2), farm animal (35), primate (1), other (1: not stated)

### Changes from abstract to publication

Changes in reporting between the abstract and publication are given in Table 2. The reported methodological quality of publications was poor: randomized in 24 (39%; method of randomization and allocation concealment not reported), blinding in 4 (6%; it was unclear for which outcomes blinding occurred in all of these), sample size calculation in 0 (0%), primary outcome stated in 3 (5%), numbers given with denominators in results 21 (34%), and number of animals stated in 35 (56%; median 20, IQR 14–35, range 5–125, total 993). Changes in reporting between abstract and publication included: from non-randomized in the abstract to randomized in the publication for 12 (19%), from no mention of blinding to blinding for some outcomes in 4 (6%), from having a primary outcome in the abstract to no primary outcome stated in the publication for 10 (16%; this is for 10/13, 77% of the abstracts that stated a primary outcome), from main outcomes being negative in the abstract to being positive (or excluded) in the publication for 5 (8%), and from main outcomes being non-statistically significant (or no statistical significance stated) in the abstract to being statistically significant in the publication for 23 (37%). In the publications the main outcomes reported from the abstract were positive for 62 (100%), and statistically significantly so for 58 (94%).

**Table 2:** Changes in reporting from abstract to subsequent publication of animal research presented at an international critical care conference

Variable	Prevalence in abstracts n = 100	Prevalence in publications n = 62	Change from abstract (A) to publication (P)
Randomized	27 (27%)	24 (39%)	12/62 (19%): non-R in A; R in P
Method of randomization	0 (0%)	0 (0%)	0 (0%)
Allocation concealment	0 (0%)	0 (0%)	0 (0%)
Blinding (possible)	0 (0%)	4 (6%)	4/62 (6%): no mention in A; blinding of some outcomes in P
Sample size calculation	0 (0%)	0 (0%)	0 (0%)
Primary outcome stated	26 (26%)	3 (5%)	10/62 (16%): 9 stated in A, not stated in P; 1 stated primary outcome was different between A and P
Numbers with denominators	6 (6%)	21 (34%)	17/62 (27%): no denominators in A; denominators in P
Main outcomes positive	90 (90%)	62 (100%)	5/62 (8%): negative in A; positive (or excluded) in P
Number of animals stated^a^	47 (47%)	35 (56%)	13/62 (21%): in the P the number was smaller in 3 (5%) and larger in 10 (16%)
Statistically significant result of main outcomes^b^	55 (55%)	58 (94%)	23/62 (37%): not significant (or not stated) in A; significant in P

*A* abstract, *P* publication, *R* randomized

^a^ In abstract: median 18 [IQR 11–24] (range 1–60), total 957 animals used. In publication: median 20 [IQR 14–35] (range 5–125), total 993 animals used. When smaller in publication: by 3, 4, and 6 animals. When larger in publication: by median 14 [IQR 5–25] range 4–54, total 213 animals. Reasons for change in number were due to: new control group (1), different numbers in both control and intervention group (9), different numbers in the only group in the study (1), new reason animals required (2), or not clear (1)

^b^ In the 23 that changed in statistical significance from A to P: the animal numbers did not change in 5 [these numbers did change in 4 (larger number in 2, and smaller number in 2), and change could not be determined in the rest because numbers were not stated in P in 9, and were not stated in the A in 5]; the main outcomes changed in 2; and denominators changed in 3 [change could not be determined in 19 others because denominators were not reported; thus we could be sure that denominators did not change in only 1]

### Post-hoc predictors of subsequent publication

Given the unexpected changes from abstract to publication, we determined predictors for subsequent publication using updated numbers combining abstract and publication (when available) data (Table 1). The methodological variables of randomization or blinding did not predict publication, but finally having main outcomes being positive (p = 0.003), and finally having statistically significant main outcomes (p < 0.001) were predictors of publication.

### Post-hoc comparison of oral versus poster presentations

Given that oral presentation was a possible predictor of subsequent publication (p = 0.025), we compared oral to poster presentations on quality of abstracts and publications (Table 3). Orally presented abstracts were less likely to report animal numbers (p = 0.02) and randomization (p = 0.06) than poster presented abstracts, and orally presented abstracts that were subsequently published remained less likely to report animal numbers and randomization than poster presentations that were subsequently published.

**Table 3:** Post-hoc comparison of oral versus poster abstracts and publications

Abstract variables	Oral (n = 16)	Poster (n = 84)	p value
Research location in North America	14 (88%)	57 (68%)	0.14
Methodological quality			
Randomized	1 (6%)	26 (31%)	0.06
Primary outcome given	2 (13%)	24 (29%)	0.23
Numbers with denominators	0 (0%)	6 (7%)	0.59
Ethical quality			
Highest species rodent	13 (81%)	48 (57%)	0.07
Number of animals stated	3 (19%)	44 (52%)	0.02
Number of animals used	28 (SD 17)	29 (SD 12)	0.27
Outcomes			
Main outcomes positive	16 (100%)	74 (88%)	0.36
Statistically significant result	9 (56%)	46 (55%)	0.95
Type of animal model			
Sepsis	8 (50%)	52 (62%)	0.41
Drug used	8 (50%)	53 (63%)	0.40
Surgery	11 (69%)	51 (61%)	0.59

Publication variables	Oral published (n = 14)	Poster published (n = 48)	p value
Methodological quality			
Randomized	1 (7%)	23 (48%)	0.006
Blinded	0 (0%)	4 (8%)	0.57
Primary outcome given	0 (0%)	3 (6%)	0.99
Numbers with denominators	3 (21%)	18 (38%)	0.35
Ethical quality			
Number of animals stated	4 (29%)	31 (65%)	0.03
Number of animals used	46 (SD 55)	26 (SD 19)	0.53
Outcomes			
Main outcomes positive	14 (100%)	48 (100%)	0.99
Statistically significant result	13 (93%)	45 (94%)	0.99
Journal factors			
Journal impact factor	5.1 (SD 3.2)	5.3 (SD 5.3)	0.92
Months to publication	31 (SD 23)	20 (SD 14)	0.12

Comparisons made using Fisher’s Exact or Chi square test, or independent samples student t test, as appropriate

### Post-hoc comparison of abstracts and publications according to journal impact factor

Given the lack of methodological differences between published and unpublished abstracts, as suggested by a reviewer, we compared quality between lower (at or below median) and higher (above median) journal impact factor publications. There were no statistically significant differences in abstract quality between those subsequently published in lower vs higher impact journals (Table 4). The only statistically significant difference in quality between publications in lower vs higher impact journals was in blinding for some outcomes, which was used in 0% of lower impact publications and 4 (13%) of higher impact publications (p = 0.049).

**Table 4:** Post-hoc comparison of abstracts that were subsequently published in lower versus higher impact journals

Potential predictor variable	Published in lower impact (n = 32)	Published in higher impact (n = 30)	p value^a^
Type of presentation			
Oral (vs. poster) presentation	7 (22%)	7 (23%)	0.99
Research location in North America	17 (53%)	23 (77%)	0.07
Methodological quality variables			
Randomized	7 (22%)	10 (33%)	0.40
Blinded	0 (0%)	0 (0%)	–
Primary outcome given	6 (19%)	7 (23%)	0.76
Numbers with denominators	1 (3%)	3 (10%)	0.35
Number of animals stated	14 (44%)	16 (53%)	0.61
Sample size calculation	0 (0%)	0 (0%)	–
Ethical quality variables			
Highest species rodent	20 (63%)	15 (50%)	0.32
>19 animals used	6 (19%)	9 (30%)	0.30
Outcome variables			
Main outcomes positive	28 (88%)	29 (97%)	0.36
Statistically significant result	17 (53%)	18 (60%)	0.59
Type of animal model			
Sepsis	9 (28%)	18 (60%)	0.02
Drug used	14 (44%)	10 (33%)	0.44
Surgery performed	12 (38%)	13 (43%)	0.80
Animals stated to be killed	19 (59%)	15 (50%)	0.61

^a^Comparisons made using Fisher’s Exact or Chi square test. For published articles, the journal impact factors were: mean 5.2 (SD 4.9), median 4.5 [IQR 2.4, 7.4], range 0.02–30.36; 5 articles were published in journals with impact factor >10

## Discussion

There are several important findings from this study. First, only 62% (95% CI 52–71%) of AR abstracts presented at an international critical care conference, the Society for Critical Care Medicine 2008 Conference, were subsequently published. This means that much AR that is ready for presentation to peers does not contribute to the biomedical literature. Second, predictors of publication were oral presentation, and finally having positive outcomes and statistically significant results. Orally presented abstracts were not of higher methodological or ethical quality. These predictors confirm that much of the reason for non-publication is due to PB, which leads to a biased representation of biomedical research in the published literature. This is the first study of which we are aware to directly confirm PB in the AR literature. Third, the reported methodological quality of abstracts and publications is poor, particularly due to no mention of method of randomization, allocation concealment, or sample size calculation, and infrequent mention of randomization, primary outcomes, number of animals used, and numbers with denominators provided for results. This suggests that even published AR has poor internal validity. That the methodological quality did not differ between abstracts and publications suggests that this quality was not a determinant of acceptance for publication. Moreover, quality did not differ between abstracts subsequently published in higher versus lower impact journals. Finally, the change in reporting from abstract to publication is of concern. Although infrequent, the increase in reporting of randomization (by 19%) and blinding (by 6%), the decrease in reporting of a primary outcome (by 16%), and the increase in positive findings (by 8%) and statistically significant findings (by 37%) suggest that in published AR there may be selective analysis and outcome reporting bias that aims to find reportable results. The change in numbers of animals reported from abstract to publication in 13/35 (37%) of those studies reporting animal numbers [in the publication the number was smaller in 3 (9%) and larger in 10 (29%)] also suggests the possibility of checking the data during the ongoing study until a significant difference is found (i.e., repeated interim analyses of data as they accumulate). These changes were not significantly less frequent in publications in higher vs. lower impact factor journals (Table 5).

**Table 5:** Post-hoc comparison of reporting in publications, and changes in reporting from abstract to publication, according to journal impact factor

Variable	Prevalence in lower impact n = 32	Prevalence in higher impact n = 30	p value of comparison	Change from abstract (A) to publication (P)
Randomized	12 (38%)	12 (40%)	0.99	
Change from A to P	8 (25%)	4 (13%)	0.25	All 12: from non-R in A, to R in P
Method	0 (0%)	0 (0%)	–	–
Allocation concealment	0 (0%)	0 (0%)	–	–
Blinding (possible)	0 (0%)	4 (13%)	0.049	
Change from A to P	0 (0%)	4 (13%)	0.049	All 4: from no mention in A, to blinding of some outcomes in P
Sample size calculation	0 (0%)	0 (0%)	–	–
Primary outcome stated	3 (9%)	0 (0%)	0.09	
Change from A to P	3 (9%)	6 (20%)	0.24	All 9: state in A, to not stated in P
Numbers with denominators	8 (25%)	13 (43%)	0.13	
Change from A to P	6 (19%)	10 (33%)	0.19	All 16: no denominators in A, to denominators in P
Main outcomes positive	32 (100%)	30 (100%)	–	
Change from A to P	4 (13%)	1 (3%)	0.19	All 5: negative in A, to positive or excluded in P
Number of animals stated^a^	17 (53%)	18 (60%)	0.59	
Change from A to P	6 (19%)	7 (23%)	0.66	From A to P the number was smaller in 3 (9%) and larger in 10 (29%)^a^
Statistically significant result of main outcomes	30 (94%)	28 (93%)	0.95	
Change from A to P	13 (41%)	10 (33%)	0.55	All 23: not significant (or not stated) in A, to significant in P^b^
Months to publication	27 (SD 17)	18 (SD 16)	0.02	–

Comparisons made using Fisher’s Exact or Chi square test, or independent samples student t test, as appropriate

*A* abstract, *P* publication, *R* randomized

^a^ In the 13 that changed in animal numbers from A to P: in the lower and higher impact P the number was smaller in 1 (by n = 4) and 2 (by n = 3 and 6), and larger in 5 (by n = 4, 14, 36, 52, 54) and 5 (by n = 4, 5, 7, 22, 25) respectively

^b^ In the 23 that changed in statistical significance from A to P, in the lower and higher impact P respectively: the animal numbers did not change in 4 and 1 (p = 0.19) [these numbers did change in 2 and 2 (larger number in 2, and smaller number in 2), and change could not be determined in the rest because numbers were not stated in P in 6 and 3, and were not stated in the A in 1 and 4]; the main outcomes changed in 2 and 0 (p = 0.16); and denominators changed in 1 and 2 (p = 0.52) [change could not be determined in 19 others because denominators were not reported in 11 and 8; thus we could be sure that denominators did not change in 1]

These findings are compatible with previous research. In clinical research, PB in reporting findings from abstracts presented at conferences has been well recognized; only about 45% of abstracts are subsequently published, and predictors of subsequent publication include positive and statistically significant findings [2–4]. In addition, for clinical research, the quality of poster and orally presented abstracts has been similar [26]. Previous indirect assessments of PB in AR have suggested small study bias, and excess significance bias [10–18]. A survey of animal researchers in the Netherlands found that respondents estimated only 50% (95% CI 32–70%) “of ethics-approved experiments performed in experimental animal research is published,” well within our 95% CI of publication rate [27]. Many previous reviews have found poor methodological quality of reported AR, including in the field of critical care [28–33]. Although selective analysis and outcome reporting bias, and data “peaking” to find significant results (instead of pre-defined sample sizes being calculated) have been suspected in AR by commentators, this is the first confirmation of this occurring as far as we are aware [6, 7, 18, 34].

These findings are important for several reasons. In clinical research PB poses a serious threat to validly assessing the effectiveness of new therapies in systematic reviews; research subjects have participated in a study without (or even negatively) contributing to science; and those who make health care decisions are faced with a biased subset of scientific evidence [2–4]. PB in pre-clinical AR is important for the same reasons; indeed, AR is often used to guide decisions for further AR and for human clinical research, and the ethical justification for AR is exactly this contribution to making decisions of benefit for human clinical research and treatment [2, 6, 7, 9]. The finding of PB in AR suggests that the valid extrapolation of findings from the AR literature to inform research and treatment in humans is seriously threatened. There are several important implications. First, strategies to combat PB are a priority. For example, funding agencies that support AR could tie funding to subsequent full publication of results, and academic institutions that employ scientists doing AR could tie full publication of results to academic review [2]. In addition, journal editors and referees have a responsibility to publish negative results, if of good quality. Second, strategies to identify PB are a priority, for example, international registration of AR protocols [2, 6, 7]. Third, attention to methodological quality requirements in funding, ethics review, and academic review decisions is a priority [28–33]. For example, if sample size calculations and statistical design for primary outcomes are required in protocols and grant applications, this can prevent data dredging, selective outcome reporting bias, ongoing recruitment aiming to obtain statistically significant outcomes, and other biases [35].

There are limitations to this study. First, it is possible that some abstracts were published but this was not detected by our search strategy. Second, it is also possible that methodological quality was better than reported, particularly in abstracts where space limitations are restrictive. We believe that this explanation is problematic. Optimal methods of randomization, allocation concealment, and blinding (of the experiments and assessment of their outcomes) are time consuming and expensive to implement. Randomization, blinding, sample size calculation for a pre-specified primary outcome (limiting “sampling to a foregone conclusion” and multiple statistical testing for post-hoc outcomes), and describing research subject numbers (including attrition or exclusion of animals) markedly improve the quality, validity, and reproducibility of even preliminary experimental results. Not reporting this information can make published findings unreliable, regardless of whether the information was in fact known to the authors. This is why guidelines for reporting clinical research in conference abstracts include these points in their minimal standards [36–38]. This is also in the spirit of the ARRIVE guidelines, meant to ensure publications are “’fit for purpose,’ meaning that incomplete reporting of relevant information effectively renders many publications of limited value as instruments to inform policy or clinical and scientific practice… unusable due to poor reporting.” [22] These points are also in the “core set of research parameters” in the US National Institute of Neurological Disorders and Stroke workshop statement, those “that should be addressed when reporting the results of animal experiments… that would allow informed judgment about the findings” [32]. Third, the sample size of 100 abstracts and 62 publications limits study power to detect predictors of publication, or of publication in lower versus higher impact journals. The analyses of predictors should be considered exploratory only, particularly given the multiple statistical testing. Fourth, this study is limited to abstracts reported at one international conference, and in the field of critical care, and may not generalize to other conferences and fields. Finally, we did not assess the quality of statistical methods used; however, this may not be possible given that most animal research publications examine statistically well over 20 outcomes [28].

Nevertheless, the strengths of this study mitigate some of these concerns. This study was done with clear data definitions and a data collection form, and with a clear rigorous search strategy used by two authors independently. In addition, the field of critical care AR usually involves quite invasive management of animals (e.g., invasive procedures done for animal monitoring, and invasive study interventions such as surgery and ventilation), and therefore might be expected to be most likely to be reported and done to high standards [28]. Finally, the results are similar to many previous reports suggesting PB in clinical and AR, and reports regarding the methodological quality of AR [2–4, 6, 7, 10–18, 27–33]. Our method of determining PB, that is, by determining subsequent publication of data presented in abstract form at an international conference, has been widely accepted in clinical research [3, 4].

## Conclusions

A direct assessment of subsequent publication of AR presented as abstracts at an international critical care conference confirms PB is prevalent. This is of concern because it suggests that published AR is a biased representation of research findings, and that animals are harmed without benefit, and with potential harm (misleading literature reviews), to humans. If AR has any chance of translating to human medicine addressing this problem of PB must be a high priority for researchers, funders, journals, and clinicians alike [39, 40].

## Additional files

**Additional file 1.** The case report form for the study.

**Additional file 2.** The study manual. This file provides the definitions used, and the details of methods for performing this study.

## Abbreviations

AR: animal research
PB: publication bias
