# Supplementary material for: Publication bias in animal research presented at the 2008 Society of Critical Care Medicine Conference
Source: BMC Res Notes. 2017 Jul 7;10:262. doi: 10.1186/s13104-017-2574-0 (PMC5501347; doi:10.1186/s13104-017-2574-0)
Supplement: Supplementary file 1 — Additional file 1. The case report form for the study. [file 13104_2017_2574_MOESM1_ESM.pdf]

Publication bias in animal research presented at the 2008 Society of Critical Care Medicine Conference

Additional File 1 (Joffe):

Data Collection Form for the study.

Journal: BMC Research Notes

Authors: Una Conradi BSc candidate[1], Ari R Joffe MD, FRCPC[2]

Affiliations: 1. University of Alberta, Faculty of Science; 2. University of Alberta, Faculty of Medicine, Department of Pediatrics, Stollery Children's Hospital, Edmonton, Alberta, Canada, and University of Alberta, John Dossetor Health Ethics Center.

Corresponding Author: Ari R Joffe MD; 4-546 Edmonton Clinic Health Academy; 11405 87 Avenue; Edmonton, Alberta, Canada; T6G 1C9. Phone: 780 2485435. Email: ari.joffe@ahs.ca

Fax: 888 7901283

**Authors**	
**Title**	
**KEYWORDS**	1.<br>2.
Abstract	
Continent of origin?	a. Asia<br>b. North America<br>c. Europe<br>d. Africa<br>e. South America<br>f. Australia/NZ<br>g. Unknown
Oral vs poster presentation	a. Oral<br>b. Poster
Randomized?	a. Yes<br>b. No
If randomized: Method of Randomization	a. Described and true randomization<br>b. Described and not true randomization<br>c. Not described
If randomized: Allocation Concealment?	a. Described and adequate<br>b. Described and inadequate<br>c. Not described
Blinded?	a. All outcomes<br>b. All subjective outcomes<br>c. Not blinded<br>d. Unclear blinding<br>e. No mention of blinding
Sample size calculation?	a. Yes<br>b. No<br>If Yes: number ____________
Primary outcome described	a. Yes<br>b. No
If described: what are the primary outcome(s): up to 2	
Numbers with denominators in outcomes?	a. Yes<br>b. No
Highest species of animal in study?	
Number of animals in results and/or methods	a. Yes<br>b. No<br>If Yes: number________
Main outcomes?	a. Positive<br>b. Negative
Statistically significant result for main outcomes?	a. Yes<br>b. No<br>c. Not stated
Condition being modeled (answer all that	a. sepsis;

apply)	b. drug used;<br>c. surgery performed;<br>d. animals killed;<br>e. none of these
Article	
Published? (Y/N)	a. Yes.<br>b. No
**If Published: answer the questions below.**	
Title	
Months to publication?	______________
Randomized?	a. Yes<br>b. No
Change compared to abstract?	a. Yes<br>b. No
If randomized: method of randomization?	a. Described and true randomization<br>b. Described and not true randomization<br>c. Not described
If randomized: change in method of randomization from abstract?[a]	a. Yes<br>b. No<br>c. Not applicable
If randomized: Allocation concealment.	a. Described and adequate<br>b. Described and inadequate<br>c. Not described
If randomized: Change in allocation concealment from abstract?[a]	a. Yes<br>b. No<br>c. Not applicable
Blinded?	a. All outcomes<br>b. All subjective outcomes<br>c. Not blinded<br>d. Unclear blinding<br>e. No mention of blinding
Change in blinding from abstract?	a. Yes<br>b. No
Sample size calculation?	a. Yes<br>b. No<br>If Yes: number________
Change in sample size calculation from abstract?[b]	Stated:<br>a. Yes<br>b. No<br>If stated in both: number changed:<br>a. Yes<br>b. No
Primary outcome stated?	a. Yes<br>b. No
Change in primary outcome from abstract?[b]	Stated a primary outcome:<br>a. Yes<br>b. No<br>If a primary outcomes stated in both: different primary outcome:

	a. Yes<br>b. No
Numbers with denominators?	a. Yes<br>b. No
Change from abstract?	a. Yes<br>b. No
Main outcomes from abstract	a. Positive<br>b. Negative
Change from abstract?	Different main outcomes:<br>a. Yes<br>b. No<br>Different pos/neg from abstract:<br>a. Yes<br>b. No
Number of animals used?	Stated:<br>a. Yes<br>b. No<br>If yes: number _______<br>If numbers stated in abstract and paper:<br>a. Smaller [by what number______]<br>b. Larger [by what number_______]<br>c. No change
If numbers are different, what is the reason?	a. no control group mentioned in the abstract, but control group mentioned in the publication<br>b. control group mentioned in the abstract, but control group not mentioned in the publication<br>c. different numbers in both control and intervention groups<br>d. different numbers in one of the groups (control or intervention), but not both<br>e. different numbers in the only group in the study<br>f. new reason animals were required in the study<br>g. not clear
Statistically significant result for main outcomes from abstract?	a. Yes<br>b. No<br>c. Not stated
Did significance change for main outcomes from abstract?	a. Yes<br>b. No
