# Supplementary material for: Publication bias in animal research presented at the 2008 Society of Critical Care Medicine Conference
Source: BMC Res Notes. 2017 Jul 7;10:262. doi: 10.1186/s13104-017-2574-0 (PMC5501347; doi:10.1186/s13104-017-2574-0)
Supplement: Supplementary file 2 — Additional file 2. The study manual. This file provides the definitions used, and the details of methods for performing this study. [file 13104_2017_2574_MOESM2_ESM.pdf]

Publication bias in animal research presented at the 2008 Society of Critical Care Medicine Conference

Additional File 2 (Joffe):

Instruction Manual for the study.

Journal: BMC Research Notes

Authors: Una Conradi BSc candidate[1], Ari R Joffe MD, FRCPC[2]

Affiliations: 1. University of Alberta, Faculty of Science; 2. University of Alberta, Faculty of Medicine, Department of Pediatrics, Stollery Children's Hospital, Edmonton, Alberta, Canada, and University of Alberta, John Dossetor Health Ethics Center.

Corresponding Author: Ari R Joffe MD; 4-546 Edmonton Clinic Health Academy; 11405 87 Avenue; Edmonton, Alberta, Canada; T6G 1C9. Phone: 780 2485435. Email: ari.joffe@ahs.ca

Fax: 888 7901283

CRF Manual for Animal (Mammals) Study Review: we include all experiments involving mammals.

Experiment: a procedure for collecting scientific data on the response to an intervention in a systematic way to maximize the chance of answering a question correctly or to provide material for the generation of new hypotheses. It involves some treatment or other manipulation that is under the control of the experimenter, and the aim is to discover whether the treatment is causing a response in the experimental subjects (mammals in this study) and/or to quantify such response. As opposed to observational study that is to find associations between variables that the scientist cannot usually control.

ABSTRACT:

Author:
-write the authors' names [Last name, initials]

Title:
-write the title of the abstract

Keywords:
-these will be used to search for whether the study was published. We are pre-specifying these prior to the search.
- Keyword choices are MeSH terms, and can be searched on the following website: https://www.nlm.nih.gov/mesh/MBrowser.html

Examples (alphabetically) that may be common include (use the most general of these):
Acute lung injury;
Abdominal compartment syndrome;
Apoptosis;
Blood transfusion;
Brain death;
Brain injuries, traumatic (use this for head trauma);
Cardiopulmonary resuscitation (use this for cardiac arrest);
Circulatory Arrest, Deep Hypothermia Induced
Cytokines;
Endotoxemia;
Hyperglycemia;
Hypothermia;
Ischemic postconditioning;
Liquid ventilation;
Multiple organ failure;
Neutrophils;
Pancreatitis;
Parenteral nutrition;
Pneumonia;
Reperfusion injury (use this for ischemia-reperfusion);
Respiration, artificial (use this for mechanical ventilation);
Respiratory distress syndrome, adult (use this for ARDS);
Respiratory syncytial viruses (use this for RSV);

Resuscitation (use this for volume administration strategies);
Saline solution, hypertonic;
Seizures;
Sepsis;
Shock;
Shock, hemorrhagic;
Stem cell transplantation;
Stem cells;
Transplants (use this for organ transplants);
Ventilator-Induced lung injury
Wounds and injuries (use this for ‘trauma’)

Continent of Origin:
-choose which continent the first author is associated with, or if not stated for each author, of the center where the work was done.
-this is for descriptive purposes, to determine how generalizable the results are.

Oral vs Poster:
-this is stated at the top of the page of each set of abstracts.
-this is because some reviews in human research suggest oral presentations are more likely to be published.

Randomization:
-described as ‘randomized’ in the abstract, using the word ‘randomized’.
-this is because some reviews in human research suggest randomized studies are more likely to be published.

Method of Randomization:
-assigned (allocated) to groups on the basis of chance, at random, in a way that cannot be predicted.
-Non-randomized methods of allocation: alternation, by dates or days of the week, picked ‘randomly’ out of the cage, historical controls (animals that had similar care without the intervention at an earlier time), other. These are not random (are deterministic allocation).
-Randomization procedure that are acceptable include a description of how the sequence of randomization was generated (an unpredictable allocation sequence). Examples: random number table, computerized random number generator, other.
-this is to determine how often true randomization occurs, and if this is a predictor of subsequent publication.

Allocation concealment:
-concealment of the allocation sequence from those enrolling animals until assignment to the group occurs (i.e. not known until the animal is enrolled into the study). Prevents selection bias by protecting assignment sequence until allocation. Methods include- a third-party (not involved in doing the experiment) tells what group that animal goes to (e.g., pharmacy, central telephone randomization, person independent of the study), or sealed sequentially numbered identical opaque containers/envelopes.
- this is a strong measure of methodological quality and may be associated with subsequent publication.

Blinding: also called masking.

-prevents performance and ascertainment bias.
-The group the animal is in is not known by the humans involved during the experiment. There are various phases of blinding: at disease induction [those inducing the disease (eg. injecting the bacteria); if the disease induction occurs before randomization, then it is not applicable];during intervention/treatment [those carrying out the intervention (eg. giving antibiotics, drug, or surgical treatment) and caring for the animals during the experiment]; and during outcome assessment: [those collecting the data or scoring the outcome].
-for this study we will *only assess whether there is blinding during outcome assessment*, and this is particularly important for subjective outcomes (scoring system, histology or pathology, etc). Objective outcomes are those that are not open to interpretation: death or survival; a measured physiologic variable at a specific time; etc.
-if 'blinding' is stated, but it is unclear whether this is for all outcome, all subjective outcomes, or just some outcomes, then respond 'unclear'.
-this is a strong measure of methodological quality and may be associated with subsequent publication.

Sample size calculation:
-should describe the primary outcome, significance level (p value, alpha value; the probability of a type I error, that is, rejecting the null hypothesis when the null hypothesis was true, a false positive finding), power (1-ß; the probability of a type II error, that is, accepting the null hypothesis when the null hypothesis is false, a false negative finding), baseline risk (the risk for the primary outcome in the control group; and standard deviation if a continuous outcome), minimally important difference (the size of a change in the primary outcome which would be important to detect).
-if a cluster trial (groups of animals are allocated to the two experimental groups, eg, allocated by cage, litter, laboratory, etc): sample size needs to be adjusted for cluster effect if the outcomes are measured at the individual animal level (i.e. outcomes not measured at the aggregated cluster level). There is non-independence of the outcome and exposures within a group of animals that are housed together or have something more in common. Need *cluster size*, *intracluster (intraclass) correlation coefficient* (the degree of correlation within clusters; the proportion of total variance of the outcome that can be explained by the variation between clusters), to calculate a *design effect*, which is multiplied by the sample size to calculate the required number of clusters. Any mention of one of the italicized words above will be considered adjusting the sample size for cluster design.
-If sample size calculation is done, then determine if the following are reported: the *minimally important difference* (the difference between groups that the study is to be powered to detect), *alpha* (the p-value), and *beta/power*. To be considered a sample size calculation, mention of these three values must be stated.
-this is a strong measure of methodological quality and may be associated with subsequent publication.

Primary outcome:
-used for sample size calculation, and the main outcome of interest that will be statistically tested. This can be a composite outcome (i.e. one outcome that is combined other outcomes: eg. survival with good neurological status is a single composite outcome). This main outcome is important to be pre-specified (should be specified before the experiment starts). There is usually only one primary outcome, but there could be two.

-if the same main outcomes are determined in several different groups, or at several different times, without one or two of these being specified as most important, the answer is: no.

-if a single (or up to two) main outcome(s) of interest is stated, then we will consider this a statement of a primary outcome.
-write down the primary outcome(s) here (to be used later for comparison to published paper).
-this is a strong measure of methodological quality and may be associated with subsequent publication.

Numbers with denominators in outcomes:
-the number of animals analyzed for each outcome should be clearly stated: i.e. denominators clear (e.g., not just a percentage given; absolute numbers are explicitly stated, and not implied so that it takes reading at several places in the manuscript to figure out the numbers exactly).
-this is important because otherwise we do not know if there were animals excluded for unclear reasons from analysis of the outcomes.
-if given for the majority of outcomes (and always including the primary outcome), then answer 'yes'. Otherwise, answer 'no'.
-this is a strong measure of methodological quality and may be associated with subsequent publication.

Highest species of animal used: all are mammals.
-examples are non-human primate > pig, dog, cat > rat, guinea pig > mouse.
-this is for descriptive purposes, to aid in determining generalizability.

Total number of animals used in the experiment.
-This is from reading methods and results sections. If not stated, or very unclear, then write 'no'. If stated or able to figure it out, write 'yes' and give the number.
-a larger sample may be more likely to be published, and if it is not, this raises greater ethical issues.

Positive and negative outcomes:
-positive outcome is a better outcome with the studied intervention (in direction that suggests benefit from intervention or in direction hypothesized before the study started).
-negative outcome is a worse outcome with the studied intervention (in direction that suggests harm from intervention, or is opposite to what was hypothesized before the study; wording may be given as 'adverse', 'worse', 'negative', 'harmful', etc, or is worded in a way that is obviously negative considering the studies objectives).
-neutral outcome is no difference in outcome between control and intervention group, and will also be considered a negative outcome.
-if there is no pre-specified primary outcome and the majority of outcomes are positive, or, if there is a pre-specified primary outcome and this is positive, then the answer is 'positive'. Otherwise, it is 'negative' or 'neutral'.
-this is part of the definition of publication bias: if positive studies are more likely to be published.

Statistically significant result for main outcomes:
-although similar to the above, this means that the positive or negative outcomes answer above is supported by statistically significant p-values (i.e. p-value <0.05).

-yes: statistically significant results shown; no: statistically the results are p>0.05 and shown; not shown: no statistical test shown to support claim.

Condition being modeled and studied:
-Sepsis model: a study to test a treatment for sepsis. The sepsis (infection) is induced in the animal model, using- injection or exposure to a bacterium, virus, parasite, or fungus; injection or exposure to a sepsis mediator (cytokine, chemokine, bacterial product or antigen, other micro-organism product or antigen); creation of an infection by bowel puncture; an infection model such as of pneumonia, peritonitis (abdominal infection), urinary infection, meningitis, wound infection, fascitis, abscess, bacteremia, etc. This is anticipated to be the largest subgroup of abstracts, and may predict publication.
-Drug used: any pharmacologic agent used as a therapeutic intervention to treat a condition (not as a treatment that was used for all groups). This could be given orally, subcutaneous, intramuscular, intraperitoneal, intravenous, etc. This is for descriptive purposes.
-Surgical procedure done: any surgical procedure (an invasive procedure with tissue incision) done antemortem, including inserting monitoring lines with use of anesthetic. This is associated with invasiveness and harm of the study, and raises greater ethical issues.
-Animals killed at end of experiment: if animals are ‘euthanized’, ‘killed’, ‘sacrificed’, ‘autopsied’, or have vital organ pathology/histology described, then we will assume at least some of the animals did not survive the experiment. A vital organ is considered any of the following: brain, spinal cord, heart, lung, liver, both kidneys, or bowel. This also raises greater ethical issues.

ARTICLE:

Published:
-by searching the first or second author, with keywords, it was determined that the experiment(s) described in the abstract were fully published.

Search strategy:
1. Search for first author ‘or’ second author; ‘and’ one key word as MeSH Term. If not found, then search as above, but using a different key word as MeSH Term.

2. First in PubMed, and if not found, then in EMBASE (Ovid).
Pubmed access:
http://www-ncbi-nlm-nih-gov.login.ezproxy.library.ualberta.ca/pubmed?otool=icauahslib
EMBASE access: use this link, and type in the ‘database’ box ‘EMBASE’
http://www.library.ualberta.ca/databases/search/index.cfm?fuseaction=title

3. Keyword choices are MeSH terms, and can be searched on the following website:
https://www.nlm.nih.gov/mesh/MBrowser.html
We will use the keywords pre-specified in the abstract data above.

Examples (alphabetically) that may be common include (use the most general of these):
Acute lung injury;
Abdominal compartment syndrome;
Blood transfusion;
Brain death;

Brain injuries, traumatic (use this for head trauma);
Cardiopulmonary resuscitation (use this for cardiac arrest);
Cytokines;
Endotoxemia;
Hyperglycemia;
Hypothermia;
Ischemic postconditioning;
Liquid ventilation;
Multiple organ failure;
Neutrophils;
Pancreatitis;
Parenteral nutrition;
Pneumonia;
Reperfusion injury (use this for ischemia-reperfusion);
Respiration, artificial (use this for mechanical ventilation);
Respiratory distress syndrome, adult (use this for ARDS);
Respiratory syncytial viruses (use this for RSV);
Resuscitation (use this for volume administration strategies);
Saline solution, hypertonic;
Seizures;
Sepsis;
Shock;
Shock, hemorrhagic;
Stem cell transplantation;
Stem cells;
Transplants (use this for organ transplants);
Wounds and injuries (use this for ‘trauma’)

Title: of the final published manuscript.

Months to publication:
Number of months from publication as the abstract (Dec, 2007), to publication in the a journal (the date the journal is published; not the acceptance date or the pre-publication date).

FROM THE METHODS SECTION OF THE PAPER:
Randomized:
-same definitions as for abstract.
Change compared to abstract:
-randomized in abstract, and not randomized as described in paper; or not randomized in abstract, and randomized as described in paper.

Method of randomization: if described as randomized in the paper
-same definitions as for abstract.
Change compared to abstract:
-change from a in abstract (described as true randomization) to b in paper (described as not true randomization); or, change from b in abstract (described as not true randomization) to a in paper (described as true randomization); or, change from c in abstract (not described) to b in paper (described as not true randomization).
-not applicable: this applies if was not randomized in abstract, and was randomized in the paper

Allocation concealment:
-same definitions as for abstract.
Change compared to abstract:
-change from a in abstract (described and adequate) to b in paper (described and inadequate); or, change from b in abstract (described and inadequate) to a in paper (described and adequate); or, change from c in abstract (not described) to b in paper (described and inadequate).
-not applicable: this applies if was not randomized in abstract, and was randomized in the paper

Blinded:
-same definitions as for abstract.
Change in blinding from abstract:
-change from a in abstract (all outcomes) to b (all subjective outcomes), c (not blinded), d (unclear blinding) or e (no mention of blinding) in paper; or, change from b in abstract (all subjective outcomes) to c (not blinded), d (unclear blinding), or e (no mention of blinding) in paper; or, change from c in abstract (not blinded) to a (all outcomes), or b (all subjective outcomes) in paper.

Sample size calculation:
-same definitions as for abstract.
Change from abstract:
1. Stated: change from a (yes) in abstract to b (no) in paper, or from b (no) in abstract to a (yes) in paper. If yes in both, then there has been no change.
2. Number changed: if sample size calculation stated in abstract. Then did the number calculated change (either higher or lower) from abstract to paper.

Primary outcome stated:
-same definitions as in abstract.
Change from abstract:
1. Stated a primary outcome: change from a (yes) in abstract to b (no) in paper, or from b (no) in abstract to a (yes) in paper.
2. Different primary outcome: if a primary outcome was stated in the abstract. Then, is the primary outcome different from in the abstract. If the abstract had two primary outcomes, then either one being used in the paper will be considered using the same primary outcome.

FROM THE RESULTS SECTION OF THE PAPER:
Number with denominators:
-same definitions as in the abstract.
Change from abstract:
-change from a (yes) in abstract to b (no) in paper; or from b (no) in abstract to a (yes) in paper.

Main outcomes from abstract:
-we had defined this in the abstract as follows: if there is no pre-specified primary outcome and the majority of outcomes are positive, or, if there is a pre-specified primary outcome and this is positive, then the answer is ‘positive’. Otherwise, it is ‘negative’.
-for this in the paper, the question is: for the main outcomes that were used to rate in the abstract, what are the results in the paper (i.e. ‘positive’ or ‘negative’).

Change from abstract:
1. Different Main Outcomes: if there was a primary outcome in the abstract, did this change in the paper; if there was not a primary outcome in the abstract, did a majority of the outcomes being analyzed in the paper change from the abstract.
2. Different pos/neg from abstract: change from a (positive) in the abstract to b (negative) in the paper, or from b (negative) in the abstract to a (positive) in the paper.

Number of animals in results/methods:
-give the number
Change from abstract: if the number was stated in the abstract. Was the number used in the paper a smaller number (by what number), larger number (by what number), or no change in number compared to the abstract.
If the number is different: Why was the number different:
-no control group mentioned in the abstract, but mentioned in the publication
-control group mentioned in the abstract, but not mentioned in the publication
[note: if one of these two, the study should not be described as randomized]
-different numbers in both control and intervention groups
-different numbers in one of the groups (control or intervention), but not both
-different numbers in the only group in the study
-a new reason animals were required in the study (e.g., change in number of animals to prepare study materials)
-not clear

Statistically significant result for main outcomes from the abstract:
-same definitions as for the abstract. This is using the outcomes reported in the abstract, and not additional ones in the paper.
Change from abstract:
-change from a (yes) in abstract to b (no) in paper, or change from b (no) in abstract to a (yes) in paper; change from c (not stated) in abstract to b (no) in paper.
